# Regional Analysis of the Brain Transcriptome in Mice Bred for High and Low Methamphetamine Consumption

**DOI:** 10.3390/brainsci9070155

**Published:** 2019-06-30

**Authors:** Robert Hitzemann, Ovidiu D. Iancu, Cheryl Reed, Harue Baba, Denesa R. Lockwood, Tamara J. Phillips

**Affiliations:** 1 Department of Behavioral Neuroscience, Oregon Health & Science University, Portland, OR 97239, USA; 2 Methamphetamine Abuse Research Center, Oregon Health & Science University, Portland, OR 97239, USA; 3 Veterans Affairs Portland Health Care System, Portland, OR 97239, USA

**Keywords:** addiction, coexpression network, connectivity, cosplicing network, gene expression, nucleus accumbens, prefrontal cortex, RNA-Seq, selective breeding, ventral midbrain

## Abstract

Transcriptome profiling can broadly characterize drug effects and risk for addiction in the absence of drug exposure. Modern large-scale molecular methods, including RNA-sequencing (RNA-Seq), have been extensively applied to alcohol-related disease traits, but rarely to risk for methamphetamine (MA) addiction. We used RNA-Seq data from selectively bred mice with high or low risk for voluntary MA intake to construct coexpression and cosplicing networks for differential risk. Three brain reward circuitry regions were explored, the nucleus accumbens (NAc), prefrontal cortex (PFC), and ventral midbrain (VMB). With respect to differential gene expression and wiring, the VMB was more strongly affected than either the PFC or NAc. Coexpression network connectivity was higher in the low MA drinking line than in the high MA drinking line in the VMB, oppositely affected in the NAc, and little impacted in the PFC. Gene modules protected from the effects of selection may help to eliminate certain mechanisms from significant involvement in risk for MA intake. One such module was enriched in genes with dopamine-associated annotations. Overall, the data suggest that mitochondrial function and glutamate-mediated synaptic plasticity have key roles in the outcomes of selective breeding for high versus low levels of MA intake.

## 1. Introduction

There is expansive literature on genome-wide transcriptome profiling results for excessive alcohol consumption and related behaviors [1,2,3,4]. In contrast, transcriptional information for methamphetamine (MA) is considerably more limited, and most studies have used microarrays for profiling. For example, Martin et al. [5] examined the effects of an acute 20 mg/kg dose of MA on rat nucleus accumbens (NAc) gene expression and identified changes in the expression of genes involved in cell-to-cell signaling, behavioral performance, and the regulation of gene expression. Piechota et al. [6], using a sensitizing regimen of MA administration in mice, induced alterations in striatal expression of several glucocorticoid-associated genes that did not persist as long as behavioral sensitization. Global miRNA expression array profiling in the NAc of rats treated with escalating doses of MA over a 15-day period, implicated miRNAs with roles in mitogen-activated protein kinase, cAMP response element binding protein, G-protein coupled receptor, and gonadotropin-releasing hormone signaling pathways [7]. In rats pretreated with MA that subsequently expressed greater conditioned reward and self-administered more MA than rats that were not pretreated, microarray analysis identified neuronal plasticity and brain development-associated genes as the major upregulated differentially expressed (DE) classes in the striatum [8]. In a foot-shock model of compulsive MA taking, *Oxt* (oxytocin) and *Cartpt* (cocaine- and amphetamine-regulated transcript protein) were among the genes upregulated in rat NAc and striatum, respectively. Finally, Cadet et al. [9] reviewed the results of transcriptional microarray studies for rats that had access to operant MA self-administration for 15 h/day on eight consecutive days, a schedule similar to the one we used to produce the MA drinking lines utilized for the research in the current studies. The authors concluded that, with regard to the effect of MA, networks that impact cellular and synaptic functions were highly represented.

The reports described above focused on the effects of MA, rather than on risk factors for MA use. Selective breeding provides a strategy for examining risk in the absence of MA exposure. Wheeler et al. [10] investigated risk genes using mice that we selectively bred for MA high drinking (MAHDR) and MA low drinking (MALDR). We used the Mouse Mood Disorder StellARay qPCR array to examine the NAc transcriptome in the MAHDR and MALDR mice. Immune pathway genes and genes that regulate apoptotic processes were well-represented among those genes that were DE [10]. A subsequent genome-wide, microarray study replicated these results. However, this study was enhanced by the inclusion of network analysis that identified a highly significant transcription factor-centric subnetwork for differential genetic risk for MA intake [11]. We know of no other studies examining the MA risk transcriptome in the absence of a history of MA exposure.

RNA sequencing (RNA-Seq) permits the determination of the presence and quantity of coding and non-coding RNAs. RNA-Seq data have been generated for many biologically significant outcomes, from drought stress in rice [12] to radiotherapy response in canines [13], the impact of chronic alcohol use in the human brain [14], and most relevant to the current analysis, the detection of genetic risk factors for mouse ethanol preference [15,16]. Compared to microarray profiling, RNA-Seq has several advantages, including a better variance structure for constructing coexpression and cosplicing networks [17,18,19]. The identification of modules of genes that are coexpressed and have conserved function provides hints about risk or effect networks that share common regulation. Cosplicing networks are distinct in both structure and function from coexpression networks, have a complementary role within the transcriptome, and can provide information about protein–protein interaction [20]. In the current study, we used RNA-Seq data to expand our analysis of the MAHDR and MALDR lines. We analyzed three brain regions in the reward pathway to address how selection impacted gene expression, network coexpression and connectivity, differential splicing (DS), differential cosplicing, and differential wiring (DW). This systems biology approach improves our understanding of shared gene regulation [21]. We also examined the expression of long intergenic noncoding RNAs (lincRNA), which are RNA molecules that are transcribed to DNA, but not translated into proteins, and have important roles as transcriptional regulators of other genes [22].

## 2. Materials and Methods

### 2.1. Husbandry

Mice were bred and housed within the veterinary medical unit of the AAALAC approved VA Portland Health Care System (VAPORHCS). Procedures were in accordance with the VA Institutional Animal Care and Use Committee and performed according to National Research Council Guidelines for the Care and Use of Laboratory Animals [23]. Mice were group housed (2–5/cage) after weaning at 21 ± 1 day of age in same sex groups. Changes in housing condition are indicated in subsections describing specific methods. Polycarbonate caging (28.5 cm × 17.5 cm × 12 cm) was used, with bed-o-cob bedding (The Andersons, Inc. Maumee, OH, USA), wire tops and filtering. Mice were maintained at an ambient temperature of 21 ± 1 °C on a 12:12 h light:dark cycle, with lights on at 0600 h. Tap water and mouse chow (Purina 5001, Animal Specialties, Woodburn, OR, USA) were available at all times.

### 2.2. Selective Breeding for MA Intake

We used mass selection procedures to generate the third replicate set of the MAHDR and MALDR lines, with 4 generations of selection to minimize random drift [24]. The originating population was a C57BL/6J × DBA/2J inbred mouse strain F2 cross, and methods were precisely as described for the replicate 1 and 2 lines [10,25], which were derived from independent sets of F2 mice of the same genetic background. The founding population of 120 F2 mice (60/sex; 53–56 days of age on the initial day of the study) was tested in a 2-bottle choice examination of water vs. MA intake. Briefly, mice were isolate-housed and given access to 2, water-filled, 25 mL graduated volumetric drinking tubes for 2 consecutive days. For the next 8 days, mice were offered a tube containing tap water and one containing MA dissolved in tap water. The concentration of MA was 20 mg/L for the first 4 days, and was increased to 40 mg/L for the next 4 days. The water tube remained on the cage for 24 h/day, while the MA-containing cylinder was available for 18 h/day. The volume consumed was determined by reading graduated markings on the tubes, and body weight was measured every 2 days and used with the volume data to calculate amount of MA consumed in mg/kg. MA intake when the 40 mg/L MA solution was available was used as the selection phenotype, and the highest consuming 26 F2 mice (half of each sex) were paired to establish the MAHDR line, whereas the lowest consuming 26 F2 mice were paired to establish the MALDR line. Selection continued in this fashion across 5 generations (S1–S5), with S4 being the last offspring generation tested for MA intake. Within each line, 56–64 mice were tested each generation (28–32/sex). We have provided justifications for the overall procedures in our previous publications [10,25].

### 2.3. Dissection of Tissue and Extraction of RNA

For the RNA-Seq analysis, we obtained brain tissue from experimentally naïve, non-MA exposed, replicate 3 MAHDR and MALDR mice from second or later litters of generation S5. Animals were 60–86 days of age at the time of tissue harvesting (mean ± SEM = 69 ± 1 day). On the day of tissue harvesting, the group-housed male mice (2–3 per cage) were moved from the colony room 4 h prior to tissue collection to allow them to acclimate to the procedure room environment. Dissection occurred between 1100 and 1300 h. Response to selection for MA intake is not sex-dependent [10,25], and male mice were used in our previous gene expression array studies under the same age and timing conditions [10,11]. Mice were euthanized by cervical dislocation and then immediately decapitated. The brain was removed using RNase-free tools, rinsed in cold saline, and then the regions of interest were bilaterally dissected out using RNase-free tools and an aluminum dissection stage. Each dissected region for each individual was placed independently into RNase-free microcentrifuge tubes containing TRIzol (Life Science Technologies, Carlsbad, CA, USA) and immediately frozen on dry ice. We originally chose this method to reduce RNA degradation, have used it for previous studies (e.g., [11]), and have consistently obtained high quality RNA. An adequate amount of RNA was obtained from the bilateral samples for each mouse, so that samples across individuals remained independent. Samples were stored at −80 °C for no longer than 1 month prior to RNA extraction. The prefrontal cortex (PFC) was removed as the medial third of a 1.5-mm slice from the anterior part of the brain just behind the olfactory bulbs. The next 1.4-mm slice included the NAc, which was taken using a micropunch fashioned from a 16-gauge blunt cut needle; the anterior commissure was used as a landmark. Next, using the superior colliculus as a landmark, the most anterior portion of the brain was removed, and the next 1-mm slice was taken, from which the ventral midbrain (VMB) was obtained. This included the remaining tissue after the ventral third of that slice and the cerebral cortex were removed. Total RNA was isolated from dissected tissue with TRIzol, using a modified single-step acid guanidinium thiocyanate-phenol-chloroform extraction method [26]. The extracted RNA was purified using a RNAeasy kit (Qiagen, Velencia, VA, USA), with RNA purity and concentration determined by NanoDrop spectrophotometry (ND-1000, Thermo Scientific, Wilmington, DE, USA). All samples submitted to the Massively Parallel Sequencing Shared Resource (MPSSR) for processing met criteria of 260/280 nm absorption ratio greater than 1.8 and greater than 100 ng of total RNA. Within the MPSSR, an RNA Integrity Number (RIN) was generated for each sample, using a Caliper LabChip GX, with an inclusion criterion of RIN > 9 for library formation. A total of 144 samples were included, 24/line/brain region.

### 2.4. RNA-Seq

Library formation (polyA+, stranded) and sequencing were performed according to Illumina’s specifications at the MPSSR. Libraries were multiplexed, 4 per lane, yielding approximately 50 million total reads per sample. Fast QC was used for checking the quality of the raw sequence data. Sequence data were then aligned using Spliced Transcripts Alignment to a Reference (STAR; [27]), allowing for a maximum of 3 mismatches per 100 bp read. Reads were uniquely aligned greater than 88%, for all samples. Reads were also aligned to known genomic features to generate counts at the gene level using featureCounts [28]. Gene expression data were imported into R and an upper-quartile normalization was performed using edgeR in Bioconductor [29]. Two samples failed normalization and were not included in subsequent analyses; the causes of the failure were not clear. Inclusion in the network analysis required a gene read density threshold of an average of greater than one count per million (CPM).

### 2.5. Network Analysis

Networks for coexpression and cosplicing were calculated as described elsewhere [15,16,20]. For both the coexpression and cosplicing networks, the weighted gene coexpression network analysis (WGCNA) algorithm was used, but the correlation matrices were different: Pearson correlations for coexpression and Mantel correlations for cosplicing. The pipeline for the cosplicing analysis is illustrated in Iancu et al. [20]. Only those genes with the top 90% of network connectivity were used for further analyses. Network modules were evaluated in enrichment in neuronal cell types by utilizing Fisher’s exact test and the data from Cahoy et al. [30]; *p*-values were corrected utilizing the Bonferroni procedure and the number of modules. The expression data have been deposited into NCBI’s Gene Expression Omnibus (GSE130254; https://www.ncbi.nlm.nih.gov/geo/query/acc.cgi?acc=GSE130254).

### 2.6. Quantitative Reverse Transcriptase-Polymerase Chain Reaction (qRT-PCR)

Two hub nodes from the connectivity analysis, which are transcripts with a large number of connections with other transcripts, were chosen for verification of DE using qRT-PCR. These 2 lincRNAs, *Gm3764* and *Gm21781*, are from a module enriched in genes associated with mRNA processing, and may drive the DE of other genes that are critical for the difference in MA intake between the selected lines (see Section 3.4.). Total RNA (1 µg) was reverse transcribed using a High-Capacity cDNA Reverse Transcription Kit (Applied Biosystems, Foster City, CA, USA). Added to each RNA sample was 2 µL of 10xRT Buffer, 0.8 µL of 25xdNTP Mix (100 mM), 2 µL of 10 × random primers, 1 µL of MultiScribe Reverse Transcriptase, and 4.2 µL of RNase-free water. The mixture was incubated at 25 °C for 10 min, 37 °C for 120 min, then 85 °C for 5 min in a PerkinElmer 9700 Thermal Cycler (PerkinElmer, Waltham, MA, USA). Predesigned TaqMan gene expression assays from Applied Biosystems (Foster City, CA, USA) were obtained for the *Gm3764* and *Gm21781* sequences, as well as for *Hprt1*, which we previously established as a good control gene for qRT-PCR for this and other mouse genetic backgrounds [31,32].

Reactions were run in triplicate, using the standard ABI 7500 Real Time PCR system (Applied Biosystems) protocol. Each reaction consisted of 4 µL of cDNA, 10 µL of 2× TaqMan Universal PCR Master Mix, 1 µL of GEX 20× primers specific for the gene of interest, and 5 µL of DEPC water. For quantification, the average Ct for the control gene (*Hprt1*) was subtracted from the Ct of *Gm3764* and *Gm21781* for each sample. Relative expression based on the ΔΔCt method (calculated as 2 to the negative power of the average expression of MALDR mouse samples minus each individual value) was used as the primary dependent variable.

### 2.7. Statistical Analyses

Statistica 13 (TIBCO, Palo Alto, CA, USA) was used to analyze the response to selection by factorial ANOVA with data grouped on line, sex, and generation. Effects were considered significant at *α* ≤ 0.05. Significant interactions were deconstructed by examining simple main effects, and post-hoc mean comparisons (Newman–Keuls test) were applied when appropriate. Heritability (*h^2^*) of level of MA intake was calculated from the slope of the regression of the selection response (*R*, based on offspring means) through S4 on the selection differential (cumulative *S*, based on population and selected parent means).

## 3. Results

### 3.1. Response to Selection

**Figure 1:**
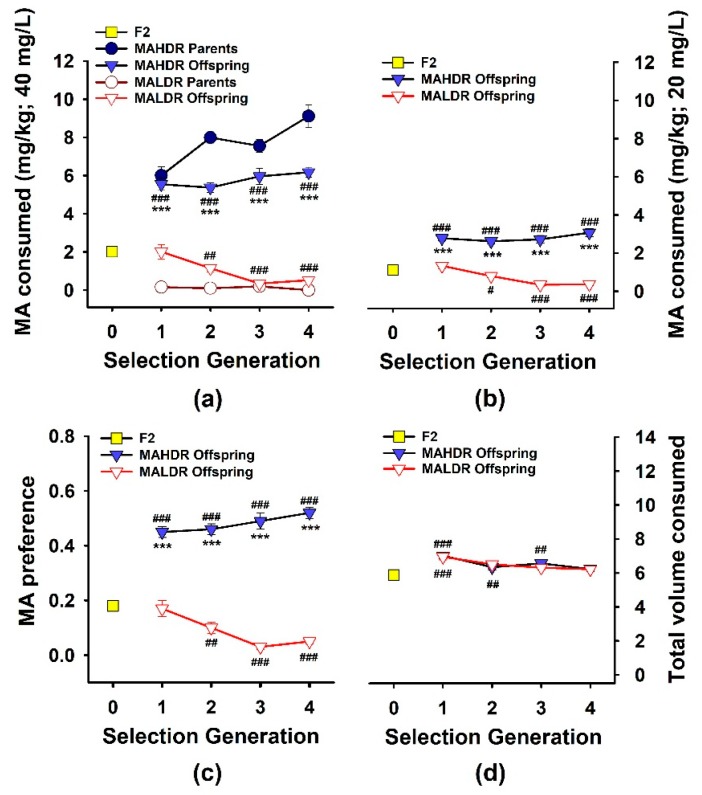
Bidirectional selective breeding results for methamphetamine (MA) consumption. Selection was based on amount of MA consumed when it was offered as a 40 mg/L concentration vs. water. (**a**) Amount of MA consumed (mg/kg) from the 40 mg/L concentration by the founding population of C57BL/6J x DBA/2J F2 mice (F2; S0), and the MA high drinking (MAHDR) and MA low drinking (MALDR) selected line parent and offspring mice over the course of selection. (**b**) Amount of MA consumed from the 20 mg/L MA concentration for the same mice. (**c**) Preference for the 40 mg/L MA concentration (mL of MA consumed/total mL fluid consumed). (**d**) Total volume consumed from the MA and water tubes (mL), during the time when 40 mg/L MA was offered. Shown are means ± SEM for the average of the second and fourth day of access to the drinking solutions for this third replicate set of MA drinking selected lines. *N* = 120 for the F2 mice, 56–64/line/generation for the MAHDR and MALDR offspring, and 26/line/generation for their parents. *** *p* < 0.001 for the difference between the MAHDR and MALDR offspring; # *p* < 0.05, ## *p* < 0.01, ### *p* < 0.001 for the difference from the F2.

Selection phenotype data for the originating population, parents selected to produce offspring for each generation, and phenotyped offspring are presented in Figure 1a. Results were consistent with previous replicates [10,25], and indicated bidirectional response to selection and differences in MA intake between the lines in all generations. For each line vs. the F2, there was a significant main effect of generation (*F*(4,362) = 11.9, *p* < 0.001 for MALDR; *F*(4,356) = 47.2, *p* < 0.001 for MAHDR). There were no significant effects of sex for these generational changes. When data for the MAHDR and MALDR lines were compared to each, there were significant generation × line (*F*(3,472) = 7.3, *p* < 0.001) and line × sex (*F*(1,472) = 5.0, *p* < 0.05) interactions. MAHDR mice consumed significantly more MA than MALDR mice at every generation, and MA intake declined across generations in the MALDR line (*p* < 0.001). The selection response of the MAHDR line was complete in the first selection generation, suggestive of a major single gene effect. Female high mice consumed more MA than male high mice (*F*(1,472) = 6.7; *p* < 0.01; mean ± SEM: 6.1 ± 0.3 and 5.4 ± 0.2 mg/kg, respectively). MA intake amounts for the female and male MALDR mice were comparable (mean ± SEM: 0.9 ± 0.2 and 1 ± 0.2, respectively).

We designed the procedure for measuring MA intake for the selection studies to avoid high levels of initial intake that might impede further voluntary consumption over time; thus, mice were offered 20 mg/L MA vs. water for four days prior to being offered 40 mg/L MA vs. water. We did not consider data for 20 mg/L MA consumption when selecting animals for breeding, but show these data in Figure 1b. The general intake patterns across generations were similar to those for the 40 mg/L MA concentration. Compared to the F2, MA intake decreased in the MALDR line across generations (*F*(4,357) = 14.4, *p* < 0.001) and increased in the MAHDR line in S1 and them remained stable (*F*(4,350) = 34.9, *p* < 0.001). MAHDR mice consumed significantly more MA from the 20 mg/L concentration than MALDR mice in all generations. For the MAHDR mice, there was a significant generation × sex interaction (*F*(4,350) = 2.4, *p* < 0.05), but a significant sex difference was present only in the S2 generation, with females consuming more MA than males (*p* < 0.05).

Preference data, calculated as MA solution consumed in mL divided by total mL consumed from both drinking tubes, are illustrated across generations in Figure 1c for the 40 mg/L MA solution. There were bidirectional changes from the F2 (*F*(4,357) = 15.3, *p* < 0.001 for the MALDR mice; *F*(4,356) = 54.2, *p* < 0.001 for the MAHDR mice) and significant differences between the lines in MA preference for all generations. There were no significant effects of sex. Finally, we examined the total amount of fluid consumed (Figure 1d) to address the possibility that selection for MA intake differences could have involved selection for differences in overall fluid consumption. For each line vs. the F2, there was a significant main effect of generation (*F*(4,357) = 9, *p* < 0.001 for the MALDR mice; *F*(4,351) = 10.4, *p* < 0.001 for the MAHDR mice). Total volume consumed was greater for both selected lines than in the F2 in some generations. There was a significant effect of sex for both lines (*F*(2,357) = 19.5, *p* < 0.001 for the MALDR mice; *F*(2,351) = 18.3, *p* < 0.001 for the MAHDR mice), with male mice consuming more total fluid than female mice. There were no significant differences in total volume consumed between the MAHDR and MALDR lines in any generation.

The heritability, *h^2^*, of this 2-bottle choice, voluntary MA intake trait was calculated through S4 to be 0.34 in the first replicate MA drinking lines and 0.35 in the second replicate lines [10,25]. The calculated *h^2^* was 0.38 in the current third replicate. Thus, approximately 38% of the variance in MA consumption in this set of MA drinking lines is attributable to genetic differences.

### 3.2. Gene Expression in the VMB, PFC, and NAc

The genes that met the expression threshold of one CPM were included in subsequent analyses [15,16]. In the VMB, PFC, and NAc, 14,102, 13,852, and 13,824 genes, respectively, met this criterion. The number of genes shared in common across all regions was 13,154, and are listed in Table S1. Dopamine neuron and receptor genes informative for specific brain regions were profiled (Table 1). As expected, levels were high for *Th, Drd2* and *Slc6a3* in the VMB, *Drd1* and *Drd2* in the NAc, and *Slc6a3* expression was below threshold in the PFC and NAc. The expression data were entered into the consensus network approach described previously [15]. Network modules within each region were color-coded; color has no meaning within or across regions. The network data were then culled to include only those genes that contributed 90% of the total network connectivity. This reduced the network sizes to 9354 genes in the VMB, 9454 in the PFC, and 8619 in the NAc (Table S2). The numbers of network modules in each of these regions were 31, 34, and 26, respectively. For each region, the enrichment in each module for genes associated with neurons, astrocytes, and oligodendrocytes is also in Table S2. For subsequent analyses, only the culled network lists were used.

### 3.3. Differential Gene Expression across Brain Regions

**Figure 2:**
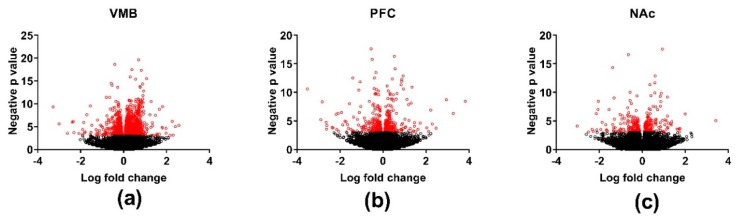
Volcano plots illustrating differential gene expression in the methamphetamine high drinking (MAHDR) and methamphetamine low drinking (MALDR) selected lines across brain regions. Brain regions analyzed were the (**a**) ventral midbrain (VMB); (**b**) prefrontal cortex (PFC); and (**c**) nucleus accumbens (NAc). Genes significantly differentially expressed between the MAHDR and MALDR lines (*p* < 0.001) are marked in red.

The genes that were significantly DE (*p* < 0.001) between the MAHDR and MALDR lines for each of the three brain regions are plotted schematically in Figure 2. Across the three brain regions, there were 3356, 189, and 49 genes that were DE between the lines at a false discovery rate (FDR) < 0.05 in the VMB, PFC, and NAc, respectively (Table S3). The DE gene lists were divided into those genes for which the MAHDR line was > the MALDR line and vice-versa. For the VMB, annotation of the genes overexpressed in the MAHDR line (*N* = 1323) revealed enrichment in categories that included translation (FDR < 6 × 10^−18^), structural constituent of the ribosome (FDR < 2 × 10^−22^), and ribosome subunit (FDR < 2 × 10^−27^) (Table S4). These genes were enriched (*p* < 0.01, corrected) in four network modules: black, brown, red and yellow. Annotation for these modules is in Table S4. None of the modules were enriched in neuronal genes. The red module was enriched in genes associated with oligodendrocytes and the yellow module was enriched in genes associated with astrocytes (Table S2). For the VMB genes overexpressed in the MALDR line (*N* = 2033), there was significant enrichment in genes associated with biological regulation (FDR < 2 × 10^−12^), biological adhesion (FDR < 2 × 10^−6^), protein kinase activity (FDR < 5 × 10^−8^), extracellular matrix structural constituent (FDR < 3 × 10^−6^), and plasma membrane (FDR < 2 × 10^−9^). These genes were significantly enriched in three network modules: green, light cyan, and turquoise (Table S3; annotation in Table S4). The turquoise module was enriched in genes associated with oligodendrocytes (Table S2). In the VMB, several network modules were moderately to strongly protected from the effects of selection, including the blue module (*p* < 0.001) (Table S3). The blue module is of particular interest, given its annotation: dopaminergic synapse (FDR < 2 × 10^−6^), acetylcholine-gated channel complex (FDR < 2 × 10^−5^), trans-synaptic signaling (FDR < 3 × 10^−7^), and G protein-coupled receptor activity (FDR < 4 × 10^−5^) (Table S4). Members of the dopaminergic synapse annotation include *Drd2* and *Slc6a3*. Table S3 indicates which of the VMB DE genes are modular hub nodes. No annotation enrichment was detected for the PFC or NAc DE genes.

### 3.4. Connectivity

Some of the hub nodes for the VMB (Table S3) were different between the MAHDR and MALDR lines in relative connectivity of ≥ 0.5 (on a scale that ranges from zero to one). For DE genes overexpressed in the MAHDR line, those differing between the lines in relative connectivity included *Atp5e*, *Sept2*, *Rap1a*, *Crot*, *Ankrd28*, *Rabggtb*, *Dzip3*, *Macrod2*, and *Bex2*. Only *Rabggtb* was a hub node in one of the modules (black) affected by selection. For the genes overexpressed in the MALDR line, there were 110 genes for which connectivity differed by ≥ 0.5 in the MALDR line, but connectivity differed by ≥ 0.5 in the MAHDR line for only 12 of these genes. Of the genes in the MALDR line for which connectivity differed by ≥ 0.5, 31 were MALDR line hub nodes. Fifteen were hub nodes in the green module (see Section 3.3), and they were *BC022960*, *Stk38*, *Srpk3*, *Rhbdf1*, *Stk38*, *Rrnad1*, *Krba1*, *Pan2*, *Nrbp2*, *Srsf11*, *Naa40*, *1700047M11Rik*, *Gm3764*, *Gm21781*, and *Clasrp*. Among these, *1700047M11Rik* (Chr 1: 182,287,990), *Gm3764* (Chr 3: 88,206,813), and *Gm21781* (Chr 10: 4,391,587) are lincRNAs. The green module is enriched in genes associated with mRNA processing (FDR < 6 × 10^−4^), regulation of RNA splicing (FDR < 6 × 10^−3^), and regulation of alternative mRNA splicing via spliceosome (FDR < 5 × 10^−2^) (Table S4).

**Figure 3:**
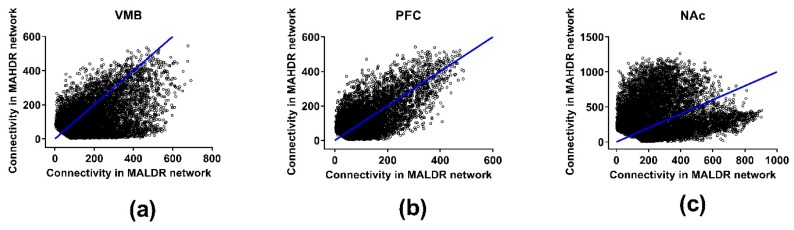
Effects of selection on total network connectivity for the coexpression networks in the methamphetamine high drinking (MAHDR) and methamphetamine low drinking (MALDR) selected lines. Connectivity is shown in (**a**) for the ventral midbrain (VMB), (**b**) for the prefrontal cortex (PFC) and (**c**) for the nucleus accumbens (NAc).

Tables S2 and S3 provide the actual total and modular connectivity data for relevant genes. The effects of selection on total network connectivity are illustrated in Figure 3. Across the network, in the VMB, average total coexpression network connectivity was significantly higher in the MALDR line (170 (MALDR) vs. 114 (MAHDR), *p* < 2 × 10^−256^), as was average modular connectivity (32 (MALDR) vs. 20 (MAHDR), *p* < 2 × 10^−151^) (Table S2). In the PFC, average total coexpression network connectivity was moderately, but significantly higher in the MAHDR line (98 (MALDR) vs. 106 (MAHDR), *p* < 2 × 10^−10^), as was average modular connectivity (16 (MALDR) vs. 19 (MAHDR), *p* < 1 × 10^−15^). In the NAc, the shift to higher coexpression network connectivity in the MAHDR line was more dramatic; average total connectivity was 259 (MALDR) vs. 368 (MAHDR) (*p* < 7 × 10^−212^), and average modular connectivity was 52 (MALDR) vs. 83 (MAHDR) (*p* < 4 × 10^−115^).

Focusing on the DE genes in the VMB, for average relative connectivity between the MALDR and MAHDR lines, there was no difference for the genes that were overexpressed in the MAHDR compared to the MALDR line (0.40 (MALDR) vs. 0.41 (MAHDR)). However, there was a significant difference in actual average intramodular connectivity (35.3 (MALDR) vs. 32.5 (MAHDR), *p* < 7 × 10^−27^). For the genes overexpressed in the MALDR compared to the MAHDR line, relative modular connectivity was significantly higher for the MALDR line (0.43 (MALDR) vs. 0.32 (MAHDR), *p* < 5 × 10^−44^), as was average intramodular connectivity (46 (MALDR) vs. 24 (MAHDR), *p* < 8 × 10^−73^).

Although the expression and DE analyses were focused on genes, there were a small group of polyA+ lincRNAs that were included in the analyses. Two of these (*Gm3764* on Chr 3: 88,206,813 and *Gm21781* on Chr 10: 4,391,587) were DE and were hub nodes in the green module (see above), and were considered to be potentially important regulators of green module coexpression. qRT-PCR was used to confirm their DE in the same VMB mRNA samples used for RNA-Seq analyses. DE was verified for *Gm21781* (*p* < 0.01; MALDR > MAHDR; 1.5-fold difference), which is a single exon lincRNA. For *Gm3764* there are 11 different transcripts annotated. We examined two of these probes and found one to be DE (*p* ≤ 0.05; MALDR > MAHDR; 1.2-fold difference).

### 3.5. Differential Wiring across Brain Regions

**Figure 4:**
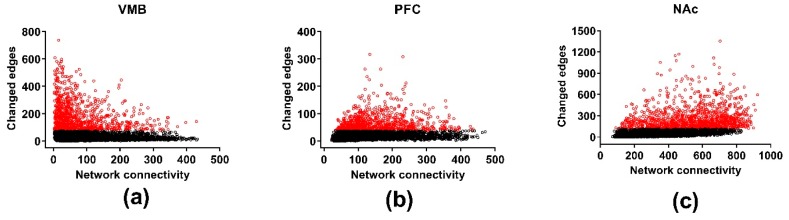
Differential wiring in the methamphetamine high drinking (MAHDR) and methamphetamine low drinking (MALDR) lines. To facilitate comparison across regions, the connectivity and number of edges are computed only for the genes shared in common across the brain regions. The numbers of changed edges for the coexpression networks are shown in (**a**) for the ventral midbrain (VMB), (**b**) for the prefrontal cortex (PFC) and (**c**) for the nucleus accumbens (NAc). Genes significantly enriched in changed edges, relative to other genes in the same network, are depicted in red.

Differential wiring (DW) measures aspects of how selection affects the gene-gene connectivity; results are illustrated in Figure 4. The threshold for DW was a change in the Pearson correlation (the edge) between two genes of ≥ 0.5. Across the three brain regions, there was significant DW for 1785, 1750, and 2063 genes (FDR < 0.05) in the VMB, PFC, and NAc, respectively (Table S5). The average number of edges affected in each of the three regions was 202, 64, and 272 for the VMB, PFC, and NAc, respectively. Significant annotations for the DW genes were for the VMB: purine ribonucleoside triphosphate binding (FDR < 2 × 10^−2^), ion binding (FDR < 7 × 10^−3^), and intracellular part (FDR < 3 × 10^−2^); for the PFC: biological regulation (FDR < 8 × 10^−4^), regulation of neurogenesis (FDR < 3 × 10^−2^), protein binding (FDR < 7 × 10^−4^), neuron part (FDR < 6 × 10^−5^), and synapse part (FDR < 4 × 10^−4^); and for the NAc: translation (FDR < 2 × 10^−28^), structural constituent of ribosome (FDR < 3 × 10^−33^), and ribosomal subunit (FDR < 6 × 10^−37^) (Table S6). The annotation for ribosomal subunits contains 18 mitochondrial ribosomal protein subunits. The annotation for synapse part includes a number of glutamate receptor related genes: e.g., *Gria1*, *Gria2*, *Grin2b*, *Grm1*, *Grm5*, and *Homer2*, and genes associated with transmitter release: e.g., *Shank1*, *Shank2*, and *Shank3*.

In the VMB, the DW genes were over-represented in five modules: green, red, and yellow (see Table S5), and greenyellow and grey60 (Tables S5 and S6). The grey60 module is enriched in genes with an astrocyte annotation (Table S2). Three modules, blue, brown, and turquoise, were significantly (*p* < 0.01 or better, corrected) protected from the effects of selection (see Table S5 for module annotation). Whereas the blue and turquoise modules were protected from DE, the brown module is enriched in DE genes. The blue module is described in Section 3.3. The brown module is enriched in annotations associated with translation (FDR < 4 × 10^−33^); the turquoise module is enriched in annotations associated with cell adhesion (FDR < 2 × 10^−2^) (Table S4). In the PFC, the DW genes were overexpressed in two modules: brown and red (see Table S6 for annotation). The brown module for this brain region is enriched in genes associated with mRNA processing (FDR < 5 × 10^−3^), RNA splicing (FDR < 5 × 10^−3^) and the spliceosomal complex (FDR < 2 × 10^−3^). The red module is enriched in annotations that included chromatin organization (FDR < 3 × 10^−3^) and nuclear part (FDR < 3 × 10^−2^). Neither module is enriched in annotations for a particular cell type (Table S2). In the NAc, there was an enrichment of DW genes in the purple and turquoise modules (Table S5); the overexpression in the turquoise module was especially notable (*p* < 1 × 10^−8^, corrected). The purple module is enriched in annotations associated with mitochondrial function, including mitochondrial protein complex (FDR < 9 × 10^−12^). The turquoise module was similar and included annotations such as ribosomal subunit (FDR < 3 × 10^−47^) that contained 18 mitochondrial ribosomal proteins. Two NAc modules, black and blue (Table S5), were significantly (*p* < 0.01, corrected) protected from DW. Of note, the black module is enriched in several annotations associated with G protein-coupled receptor signaling, that included *Drd1*, *Drd2*, and *Rgs9*. The blue module includes annotations for regulation of calcium ion-dependent exocytosis (FDR < 2 × 10^−3^), G-protein activated inward rectifier potassium channel activity (FDR < 3 × 10^−2^) and synapse part (FDR < 3 × 10^−5^).

The DW genes were also analyzed for hub nodes associated with a change of relative intramodular connectivity of ≥ 0.5. In the VMB, there were no hub nodes in the MAHDR line associated with a difference in connectivity of ≥ 0.5, compared to the MALDR line; in fact there were no genes with a difference in connectivity in the MAHDR line of this magnitude. In contrast, in the MALDR line, there were 178 genes with such a difference in connectivity, compared to the MAHDR line, including 41 hub nodes (Table S5). Eighteen of the 41 hub nodes were in the green module and included all of the DE green module hub nodes that were similarly affected (see above). New green module genes meeting criteria were *Nxf1*, *Lrrc45*, and *Anksf1*. Relative intramodular connectivity for all DW genes was significantly higher in the MALDR vs. MAHDR line (0.49 (MALDR) vs. 0.25 (MAHDR), *p* < 2 × 10^−200^). The difference in average actual intramodular connectivity between the MALDR and MAHDR lines was similarly marked (58 (MALDR) vs. 10 (MAHDR), *p* < 1 × 10^−300^).

For the DW genes in the PFC, the situation was somewhat more balanced. Seventy-four genes in the MAHDR line and 121 genes in the MALDR line had an increase in connectivity of ≥ 0.5, compared to the other line. Of these, 40 were hub nodes in the MALDR line and 18 were hub nodes in the MAHDR line. These affected hub nodes were not significantly enriched in the brown or red modules (see above). However, it was observed that the 121 affected genes in the MALDR line were significantly enriched in annotations associated with cell-to-cell signaling, including regulation of cell communication (FDR < 3 × 10^−2^). Forty-one genes were associated with cell communication (Table S5); notable genes included *Grin3a*, *Npy2r*, *Nrg1*, *Per2*, and *Pink1*. For the PFC DW genes, there was no significant difference in average relative connectivity between the MALDR and MAHDR lines (0.372 (MALDR) vs. 0.368 (MAHDR), *p* > 0.58). There was, however, a significant difference in actual average intramodular connectivity (20 (MALDR) vs. 23 (MAHDR), *p* < 8 × 10^−5^).

For the NAc, 184 DW genes in the MAHDR line and 47 DW genes in the MALDR line had a difference in connectivity of ≥ 0.5 compared to the other line. Of these, 51 were hub nodes in the MAHDR line and nine were hub nodes in the MALDR line. Twenty-two of the MAHDR line affected hub nodes were in the turquoise module and 8 were in the purple module (see above). Of these hub nodes, *Hsbp1* (purple module) and *Hspe1* (turquoise module) are heat shock proteins. Relative intramodular connectivity was significantly different between the MALDR and MAHDR lines (0.32 (MALDR) vs. 0.52 (MAHDR), *p* < 1 × 10^−137^). Actual average intramodular connectivity was also significantly different (46 (MALDR) vs. 173 (MAHDR), *p* < 7 × 10^−228^).

### 3.6. Cosplicing across Brain Regions

**Figure 5:**
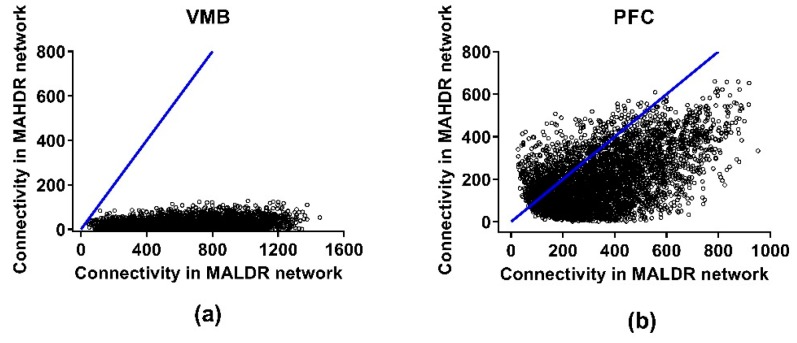
Effects of selection on total network connectivity for the cosplicing networks in the methamphetamine high drinking (MAHDR) and methamphetamine low drinking (MALDR) selected lines. Connectivity is shown in (**a**) for the ventral midbrain (VMB) and (**b**) for the prefrontal cortex (PFC). No data for the nucleus accumbens (NAc) are presented, because the data for the NAc did not follow a power law distribution; thus, it was not possible to assess differences in cosplicing connectivity.

Cosplicing networks were formed, again using the consensus network strategy. The genes entered into the analysis were the same as those meeting the threshold criteria for inclusion, i.e., one CPM. The cosplicing networks were formed as described in Iancu et al. [20], and then culled to include only those genes that contributed 90% of the total network connectivity. The data from the NAc did not follow a power law distribution; i.e., it was not possible to determine the hub nodes. The reason(s) for this failure are not clear, but further analyses of the NAc data were not performed; therefore, connectivity for cosplicing data is presented only for the VMB and PFC in Figure 5. These networks are found in Table S7 and include 5238 and 4692 genes in the VMB and PFC, respectively. Again, the network modules were color-coded with color having no meaning within or across regions. In both regions, average total cosplicing network connectivity was higher in the MALDR line, compared to the MAHDR line, by 32-fold in the VMB and 1.75-fold in the PFC (both *p* < 1 × 10^−300^).

Across the two regions, there were 3759 common genes (Table S7). The annotation of these common genes is found in Table S8. Enrichment in a number of annotations was highly significant and included cellular localization (FDR < 2 × 10^−42^), ATP binding (FDR < 5 × 10^−21^), GTPase binding (FDR < 8 × 10^−12^), Ras GTPase binding (FDR < 5 × 10^−8^), ubiquitin-like protein ligase binding (FDR < 5 × 10^−8^), intracellular part (FDR < 2 × 10^−72^), synapse (FDR < 6 × 10^−12^), postsynaptic specialization (FDR < 4 × 10^−11^), and glutamatergic synapse (FDR < 2 × 10^−10^). Genes in the glutamate annotation are listed separately in Table S8 and include *Dlg1–4*, *Dnm1–3*, *Gabbr1–2*, *Gria2–4*, *Grm3* and *Grm5*, and 3 disintegrin and metallopeptidases (*Adam10*, *Adam22*, and *Adam23*).

### 3.7. Differential Splicing across Brain Regions

Differential splicing was calculated for each gene, using a distance-based approach (see Methods) that treats each exon equally, and with only the genes in the “culled” networks entered into the analysis. Permutation analysis (*N* = 10,000) was used to determine significance. Significant (FDR < 0.05) DS was found only in the VMB for which 1341 of the 5238 genes were DS (Table S9). Annotation of the DS subset revealed a significant enrichment in genes associated with cellular component of organization or biogenesis (FDR < 6 × 10^−3^), GTPase binding (FDR < 4 × 10^−2^), and enzyme binding (FDR < 5 × 10^−2^) (Table S10). The DS genes were enriched (*p* < 0.01, corrected) in the blue, cyan, green and red network modules; the blue and green modules are significantly enriched in genes with a neuronal annotation (Table S7). Annotations for all 4 modules are found in Table S10. Notable enrichments (FDR < 0.05 or better) include blue module (regulation of signaling receptor activity, ionotropic glutamate receptor binding), green module (vesicle-mediated transport, G protein-coupled receptor binding, synapse part), and cyan module (chromatin organization, chromatin binding). The brown and turquoise cosplicing modules were protected from the effects of selection (Table S10). Annotations are found in Tables S9 and S10. Key and most significant annotations were associated with cellular organization.

**Figure 6:**
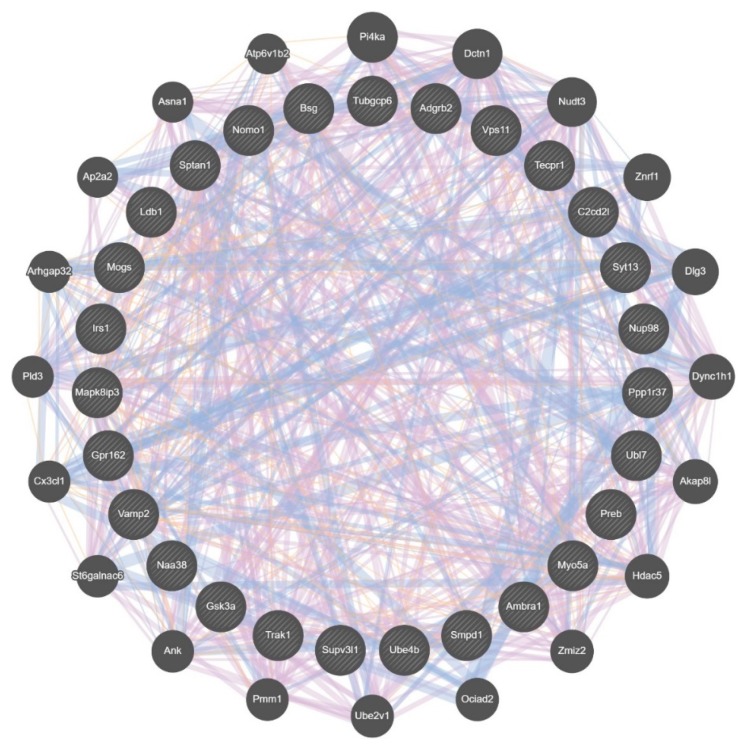
Composite gene-gene functional interaction network for differential splicing in the ventral midbrain (VMB), associated with relative genetic risk for methamphetamine (MA) intake. This network was derived from the 27 hub nodes (inner circle; symbol size has no meaning) in the MA low drinking (MALDR) line that had an increase of ≥ 0.5 in intramodular connectivity. No such genes were found in the MA high drinking (MAHDR) line. The genes in the outer circle were identified by GeneMANIA [34] as functional associates (symbol size determined by GeneMANIA, based on number of network connections). The pink lines indicate known coexpression (81%), the blue indicate known colocalization (18.5%), and the tan are predicted interactions (0.5%); these can be more clearly seen in Supplementary Figures S1–S3.

Examination of these VMB DS data for differences between the selected lines revealed that 112 genes in the MALDR line had a difference in relative intramodular connectivity of ≥ 0.5, compared to the MAHDR line; no genes in the MAHDR line had such a difference. Of the 112 MALDR line genes, 27 were MALDR line hub nodes; six of these hubs were in the blue module and seven were in the green module. The 27 hub nodes are part of a larger gene network illustrated in Figure 6; genes in this network are listed in Table S9. When further analyzed using Enrichr [33], this network was found to be associated with a protein-protein interaction network (FDR < 7 × 10^−3^), and *Grin1*, the zeta subunit of the NMDA receptor, was identified as the major hub, with key interacting members including *Dlg3*, *Dlgap4*, *Irs1*, *Myo5a*, and *Sptan1*. Overall, relative intramodular connectivity for the DS genes was significantly higher in the MALDR line compared to the MAHDR line (0.52 (MALDR) vs. 0.21 (MAHDR), *p* < 7 × 10^−262^) (Table S9). The difference in intramodular connectivity was even greater when viewed from the perspective of actual connectivity (53 (MALDR) vs. 3 (MAHDR), *p* < 1 × 10^−300^).

### 3.8. Differential Wiring of the Cospliced Genes across Brain Regions

**Figure 7:**
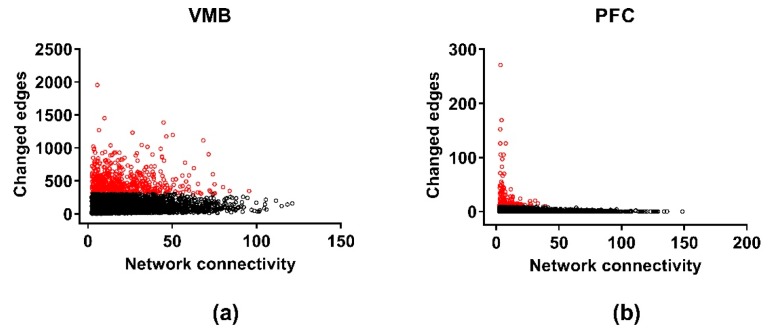
Differential wiring of the cospliced genes in the methamphetamine high drinking (MAHDR) and methamphetamine low drinking (MALDR) lines. To facilitate comparison across regions, the connectivity and number of edges are computed only for the genes shared in common across the brain regions. The numbers of changed edges for the cosplicing networks are shown in (**a**) for the ventral midbrain (VMB) and (**b**) for the prefrontal cortex (PFC). The nucleus accumbens (NAc) is not represented, because the data from the NAc did not follow a power law distribution, making it impossible to assess differential wiring for the cosplicing networks. Genes significantly enriched in changed edges, relative to other genes in the same network, are depicted in red.

For the cospliced genes, DW was calculated using a variation of the Mantel Test for all pairs of genes in the selected lines. Again, only the VMB and PFC could be included (Figure 7). As for coexpression, a difference in the pair-wise correlation between the lines of ≥ 0.5 on a scale that ranges from zero to one was taken to be a putative significant edge change, which was then confirmed by permutation testing (*N* = 10,000), and only the genes in the “culled” cosplicing networks were entered into the analyses. The lists of genes for the VMB (*N* = 1437) and PFC (*N* = 255) having significant DW, corrected for multiple testing (FDR < 0.05), are in Table S11. The average number of changed edges was significantly (*p* < 0.0001) larger in the VMB (mean ± SEM: 358 ± 6.9), compared to the PFC (mean ± SEM: 29.8 ± 2.4). Significant annotation was detected only for the DW genes in the VMB (Table S12). Notable annotations were detected for cellular metabolic process (FDR < 2 × 10^−13^), GTPase binding (FDR < 6 × 10^−3^) and mitochondrial part (FDR < 3 × 10^−10^). The GTPase binding genes are listed separately in Table S12 and include a large number of genes with Ras GTPase binding domains. The VMB DW genes were enriched in two modules—red and purple. Neither module is enriched in genes associated with a particular cell type.

For the VMB, there were 468 genes for which relative intramodular connectivity was higher in the MALDR line by ≥ 0.5; there were no genes in the reverse category. Of the 468 genes, 92 were hub nodes. Nineteen of these hub nodes were in the red module. However, overall, these hub nodes are not defined by a specific set of annotations (data not shown).

## 4. Discussion

There are several different approaches that could be used to analyze complex RNA-Seq datasets. The strategy we applied is largely network-centric and builds from previous efforts (e.g., [15,16]). Importantly, the sample sizes per group are sufficient to produce network modules of moderate to high reliability. The size and number of modules are somewhat arbitrary, but we have found that keeping the number of modules at ~25 and keeping the minimum module size at >100 genes results in annotation-rich modules. We have also taken the step of removing the extreme leaf nodes (those nodes that contribute the bottom 10% of network connectivity). This maneuver reduced the number of genes analyzed in some cases by >50% from the initial inclusion threshold. As part of our network strategy, we focus on those genes with a large change in intramodular connectivity and of those genes, we focus on the ones that are network hubs.

Table S13 provides a brief overview of 32 publications describing transcriptome/MA (or amphetamine) interactions. This survey is not intended to be all inclusive, but rather to provide the reader with some idea of progress that has been made at the genomic level. Despite these and many other studies, we believe that only Belknap et al. [11] examined how selective breeding for MA intake affected the transcriptome. However, we note that Palmer et al. [35] examined changes in the transcriptome associated with selection for MA-induced locomotor activity. The selection process used in Belknap et al. [11] was identical to that used in the current study and over several selections has been found to be associated with a large quantitative trait locus (QTL) on proximal chromosome 10 [11,36]. Additional studies identified a spontaneous single nucleotide polymorphism (SNP) in the trace amine-associated receptor 1 (*Taar1*), which nullified the function of the expressed receptor, and is a major source of increased MA intake [37,38,39,40]. Belknap et al. [11] found, in the same brain regions as those in the current study, that there were 195, 787, and 399 DE probe sets between the MAHDR and MALDR lines in the NAc, PFC, and VMB, respectively. Numerically, these data are very different from the data reported here. There are numerous reasons why microarray data [11] and RNA-Seq data may not agree (see e.g., [41]). These include: large differences in the data variance structure [19], the effect of SNPs on microarray probe set performance [42], the bias of RNA-Seq data to large and highly expressed genes (see e.g., [17,18]), differences in the expression threshold for inclusion in the subsequent data analyses, and the difficulties associated with comparing analog and binary data. Here, there is the additional difference of the considerably larger sample size for the RNA-Seq analyses (24/line/brain region), compared to the microarray analysis (5/line/brain region). Nonetheless, it seems reasonable to ask if there was overlap between the RNA-Seq and microarray datasets. To address this question, we extracted from the Belknap et al. [11] data, the genes DE in the VMB at *p* < 0.01, uncorrected. A lenient threshold was deemed appropriate, given the small sample size for the microarray data. A total of 1361 genes met this threshold, excluding duplicate probes (Table S14). Of these, 164 genes overlapped between the microarray and RNA-Seq datasets (Table S14); the overlap was better than chance (*p* < 0.01). This overlapping set is modestly enriched in genes associated with plasma membrane part (*p* < 5 × 10^−4^). Belknap et al. [11] also observed that the DE genes in all three brain regions were enriched in genes found on proximal Chr 10 and thus, near *Taar1* (Chr 10: 239203356-23921469 bp). Of the 164 common DE genes, 23 are found on Chr 10, slightly less than 200% above the expected number of 8. Of these 23, eight are found in the proximal region (0–30 Mbp). These genes are *Aig1, Fuca2, Map3k5, Pcmt1, Ppil4, Samd5, Sf3b5,* and *Ust*. All of these genes were overexpressed in the MAHDR line.

Here, we emphasize several key findings from the coexpression data. One, there were marked regional differences in the effects of selection for MA intake on the transcriptome. The data presented here illustrate that, especially from the perspective of DE, the VMB was more strongly affected than either the PFC or NAc. The significantly overexpressed VMB genes in the MAHDR line are enriched in annotations associated with translation and structural constituents of the ribosome. Both categories are enriched in mitochondrial ribosomal proteins, e.g., *Mrpl27*, *Mrpl18*, and *Mrpl49*. There is a rich literature documenting a mitochondrial role in MA neurotoxicity, including neurotoxic effects on DA neurons [43]. The data presented here suggest that mitochondrial function may also have a role in regulating MA consumption. Furthermore, *Taar1* has a role in regulating MA-induced neurotoxicity, such that *Taar1* knockout mice are more susceptible than matched wild-type mice [44]. Therefore, MAHDR mice, which are homozygous for the *Taar1* null mutation, would also be expected to be more susceptible.

The significantly overexpressed genes in the VMB of the MALDR line are enriched in genes associated with biological adhesion and the extracellular matrix (ECM). The group of genes associated with the ECM include 13 collagens (*Col4a1,4a2, 5a3, 6a6, 11a1,11a2,12a1,15a1, 16a1, 18a1, 19a1, 24a1*, and *27a1*) and seven laminins (*Lama1, a3, a4, a5, b2,* and *c1*). To our knowledge there are no reports linking collagens and laminins, specifically, and the ECM and cell adhesion molecules (CAMs), generally, to risk for MA use and addiction. However, there is substantial evidence linking the ECM and CAMs to the effects of acute and chronic MA administration [45]. For example, repeated MA treatment was associated with increased matrix metalloprotease (MMP) levels in the CNS [46]. In particular, repeated 2 mg/kg/day MA given for five days was associated with increased MMP-2 and -9 protein in the frontal cortex and NAc of rats, and an acute high dose of 40 mg/kg MA was followed by increased MMP-9 mRNA expression or increased levels of MMP-9 in multiple brain regions of mice [47,48]. In addition to these data, there is ample evidence that a variety of abused drugs (alcohol, cocaine, heroin, nicotine) can have marked effects on ECM constituents [49,50]. These data may suggest that there are some common features of the risk transcriptome, including role(s) of the ECM in synaptic plasticity [51,52,53].

Two, our network-based approach for the coexpression data coalesced on the VMB green module. The green module was one of three modules enriched in genes that are significantly overexpressed in the MALDR line. Focusing on the genes with a marked change in intramodular connectivity, 31 of the 110 genes that met criteria are found in the green module and 15 of these genes are hub nodes in the green module. The DW data also focused on hub nodes in the green module. The green module is enriched in genes associated with mRNA processing, regulation of RNA splicing and regulation of alternative mRNA splicing, via the spliceosome. The latter is a pertinent observation given that significant DS was observed only in the VMB (see below). Finally, we were able to verify the DE of 2 lincRNAs that were DE and hub nodes in the green module. Follow-up is needed to determine if they are functional and regulate coexpression of the genes within this module (see [22,54,55]).

Three, the impact on gene-network connectivity via the analysis of DW [15,16] measures an aspect of changes in correlation between all gene pairs that pass the threshold for analysis inclusion. Our threshold that the difference in correlation must be ≥ 0.5 for a changed edge is somewhat arbitrary, but has proven empirically useful in defining unique gene clusters [15,16] that are often independent from the DE gene clusters; this was especially true in the PFC and NAc. Across the three brain regions, the DW genes were clearly different. Noteworthy is the enrichment in the NAc of mitochondrial ribosomal subunits, again suggesting that mitochondrial function was affected by selection. The PFC DW genes are enriched in the largely overlapping annotations of neuron and synapse part, which is not the case in the VMB or NAc. The genes affected included a cluster of glutamate-related genes: *Gria1, Gria2, **Grin2b**, **Grin3a**, Gm1, Grm5, Homer2, **Pclo**, Shank1, **Shank2**, Shank3,* and *Slc1a2*; a cluster of GABA-related genes: *Gabbr1, Gabbr2, Gabrb1,* and *Gabrd*; two glycine receptor genes: *Glra3* and *Glrb*; a cluster of potassium channels: *Kcna3, Kcna1, Kcnc2,* and *Kcnj6*; a cluster of syntaxins: *Stx1a, Stx2, Stx3,* and *Stx6*; and two opiate receptor-related genes: *Penk* and *Oprm1*. Genes in bold and underlined are hub nodes. The broad number and functions of genes affected suggest that selection had the impact of rewiring both excitatory and inhibitory synapses (in the PFC). Two of the hub nodes, *Grin2b* and *Pclo*, have been linked to MA phenotypes. The expression of *Grin2b* was associated with context-induced reinstatement of MA seeking [56]. *Pclo*, which encodes Piccolo, a presynaptic scaffolding protein, is a regulator of behavioral plasticity and dopamine transporter internalization [57], and the reduced expression of *Pclo* augmented MA-induced behavioral sensitization, conditioned reward and synaptic dopamine accumulation in the NAc [57]. Uno et al. [58] noted that a polymorphism in *Pclo* was associated with the regulation of both dopamine and serotonin transport and was detected by GWAS as being associated with some symptoms of risk for drug dependence, including age of first exposure to MA. More generally, there is ample evidence of an association between NMDA receptors and MA pharmacology. For example, Lominac et al. [59] found that repeated low dose MA administration perturbed pre- and post-synaptic glutamate transmission in the medial PFC. Szumlinski et al. [60] concluded that an idiopathic, genetic or drug-induced hyperglutaminergic state is a key mediator of MA addiction vulnerability. In fact, the MAHDR line, a model of high genetic risk for MA intake, has higher baseline glutamate levels in both the PFC [59] and the NAc [60], compared to the MALDR line.

Four, the coexpression modules most strongly effected by selection in terms of enrichment for DE and DW genes were largely not modules enriched in neuronal genes. For example, in the VMB coexpression network, there were seven modules with a moderate to strong annotation for neuronal genes. None of these modules was significantly affected by selection. Rather, selection affected modules associated with astrocytes and/or oligodendrocytes. A similar trend was found in the NAc. We recognize that the algorithm used to determine cell type enrichment is indirect; nonetheless, the data suggest that non-neuronal cells may have had a key role in the response to selection for MA intake. These data complement the numerous observations that MA activates astrocytes and microglia [61,62].

Five, it is common to focus on those network modules that are enriched in selection-related DE, DS and DW genes. Thus, from the perspective of the VMB cosplicing data, two of the modules enriched in DS genes (blue and green) have significant enrichment in annotations associated with regulation of signaling receptor activity, ionotropic glutamate receptor binding, vesicle-mediated transport, G protein-coupled receptor binding and synapse part. However, it may be equally important to focus on those modules that are actually significantly protected from the effects of selection; i.e., modules with a significant decrease in the predicted number of affected genes. One of these protected modules was the blue module in the VMB coexpression network. This module is enriched in genes with dopamine-associated annotations, including *Drd2* and *Slc6a3*. Given that TAAR1 is thought to modulate dopaminergic activity [63,64,65], and that some effects of MA and other drugs are thought to be associated with TAAR1 through effects on dopaminergic neurons/synapses [66,67], the lack of selection effects on the blue module is seen as a key observation.

Six, stepping back from a focus on genes, pathways, and specific annotations, it is clear that selection had marked effects on coexpression network connectivity. In the VMB, network connectivity was much higher in the MALDR as compared to the MAHDR line, whereas the reverse was true in the NAc. The PFC was relatively neutral to the effects of selection on connectivity. It is tempting to speculate that the differences in connectivity align with functionality, as has recently been demonstrated for learning [68]. However, it is clear that these differences in connectivity are regionally dependent.

Our cosplicing analysis strategy [20] is biased to multi-exon and moderate to highly expressed genes, with the goal of detecting network-like patterns of exon usage. Importantly, we have observed that splicing appears to follow a power law distribution similar to that seen for coexpression, where the characteristics of each module are strongly affected by relatively few, highly connected hub nodes. We emphasized in the data analysis that there is a core set of genes common to the splicing networks in each brain region. The annotations of these common genes (cellular localization, ATP binding, GTPase binding, Ras GTPase binding, ubiquitin-like protein ligase binding, intracellular part, synapse, postsynaptic specialization, and glutamatergic synapse) appear to be independent of the background genotype; e.g., the same pattern emerges from the more genetically diverse heterogeneous stock–collaborative cross mouse [15]. The common genes tend to be large multi-exon genes with numerous known transcripts; e.g., *Dlg4*, with 22 known transcripts. This bias is reflected in the results. We have also recognized that coexpression and cosplicing cannot be completely untangled when using our genome-wide analysis strategy [20]. However, the cosplicing analysis does have a decidedly greater focus on neuronal genes and neuronal network modules.

Similar to DE, significant DS was observed only in the VMB. Some of the modules enriched in DS genes are noted above and include numerous neuronal annotations; e.g., G-protein-coupled receptor binding. Only in the MALDR line did we observe genes with an increase of ≥ 0.5 in intramodular connectivity. Of the 113 genes meeting this criterion, 27 were MALDR line hub nodes that were distributed across several cosplicing modules. We also observed that these hub nodes are part of a larger (48 genes) protein–protein interaction network (Figure 6), where *Grin1* is the key hub and some of the key interacting partners are *Dlg3, Dlgap4, Irs1, Myo5a* and *Sptan1*. Thus, and keeping with the bias noted above in mind, the cosplicing data again point to glutamate synaptic plasticity as being a key feature of understanding MA selection.

The DW analysis of the cosplicing data revealed marked effects in the VMB and modest to weak effects in the PFC and NAc. Significant annotations were detected only for the VMB DW genes and included cellular metabolic process, GTPase binding and mitochondrial part. Mitochondrial function and MA are discussed above. Although there is little published with regard to a potential role for GTPase binding, a recent paper reported that amphetamine activates Rho GTPase signaling, and that this may be a mechanism involved in amphetamine-induced internalization of the dopamine transporter, once amphetamine enters the cell [69].

## 5. Conclusions

Overall, the data presented here illustrate that bidirectional selective breeding for high and low MA consumption had marked effects on the transcriptome in brain regions that are part of the drug reward circuitry [70,71,72,73]. There could be profound changes in additional brain regions that have not yet been investigated. The effects are broad and it would be difficult to argue for the importance of a single target. However, although it is typical for selective breeding for drug-related traits to involve the cumulative effects of many genes, each with a small impact on the genetic variance in the trait, for the current MA intake selection, a single genetic change accounts for as much as 60% of the genetic variance [11]. Because this genetic change results in a null mutation that is present throughout development, it is easy to imagine that brain wiring and function could be significantly impacted. The data suggest that mitochondrial function and glutamate-mediated synaptic plasticity have key roles in the selection outcomes, but whether the differences reported here are directly linked to *Taar1* remains to be seen.

## Figures and Tables

**Table 1:** Average reads for monoaminergic genes in specific brain regions.

	*Th*	*Drd1*	*Drd2*	*Drd5*	*Slc6a3*	*Comt*
**VMB**	19235	135	3760	128	31070	3229
**PFC**	70	267	54	154	ND	2817
**NAc**	534	13086	6049	354	ND	5950

Reads reflect relative levels of expression for the methamphetamine high drinking (MAHDR) and methamphetamine low drinking (MALDR) lines, combined. *Comt*: catechol-O-methyltransferase; *Drd1*: dopamine D1 receptor; *Drd2*: dopamine D2 receptor; *Drd5*: dopamine D5 receptor; NAc: nucleus accumbens; ND: not detected; PFC: prefrontal cortex; *Slc6a3*: dopamine transporter; VMB: ventral midbrain.

## Supplementary Materials

The following are available online at https://www.mdpi.com/2076-3425/9/7/155/s1, Table S1: Genes that met the expression threshold in the MALDR and MAHDR mice and are found in all three brain regions—ventral midbrain (VMB), prefrontal cortex (PFC), and nucleus accumbens (NAc); Table S2: Coexpression network modules and associated data; Table S3: Differentially expressed (DE) genes and associated data; Table S4: GO terms for differential expression (DE) and network module descriptions; Table S5: Differential wiring (DW) and associated data; Table S6: GO terms for differential wiring (DW) and network module descriptions; Table S7: Cosplicing network modules and associated information; Table S8: Modules for cospliced genes shared by the VMB and PFC, and list of glutamatergic synapse genes; Table S9: Differential splicing (DS) in the VMB and associated information; Table S10: GO terms for differential splicing (DS) and network module descriptions; Table S11: Differential wiring (DW) results for cospliced genes; Table S12: GO terms for differential wiring (DW) of cospliced genes and network module descriptions; Table S13: MA-related transcriptome literature; Table S14: Comparison of differential expression (DE) in the VMB of MAHDR vs. MALDR lines using Affymetrix microarray 430 2.0 mouse GeneChips [11] and RNAseq data from the current study; Figure S1: Gene–gene coexpression interaction network for differential splicing in the ventral midbrain (VMB), associated with relative genetic risk for MA intake; Figure S2: Gene–gene colocalization interaction network for differential splicing in the ventral midbrain (VMB), associated with relative genetic risk for MA intake; Figure S3: Gene–gene predicted interaction network for differential splicing in the ventral midbrain (VMB), associated with relative genetic risk for MA intake. References [74,75,76,77,78,79,80,81,82,83,84,85,86,87,88,89,90,91,92,93,94,95,96,97,98] are cited in Table S13.
