# Supplementary material for: Regional Analysis of the Brain Transcriptome in Mice Bred for High and Low Methamphetamine Consumption
Source: Brain Sci. 2019 Jun 30;9(7):155. doi: 10.3390/brainsci9070155 (PMC6681006; doi:10.3390/brainsci9070155)
Supplement: Supplementary file 1 [file brainsci-09-00155-s001.zip › supplementary-for conversion/Supplementary Figure.docx]

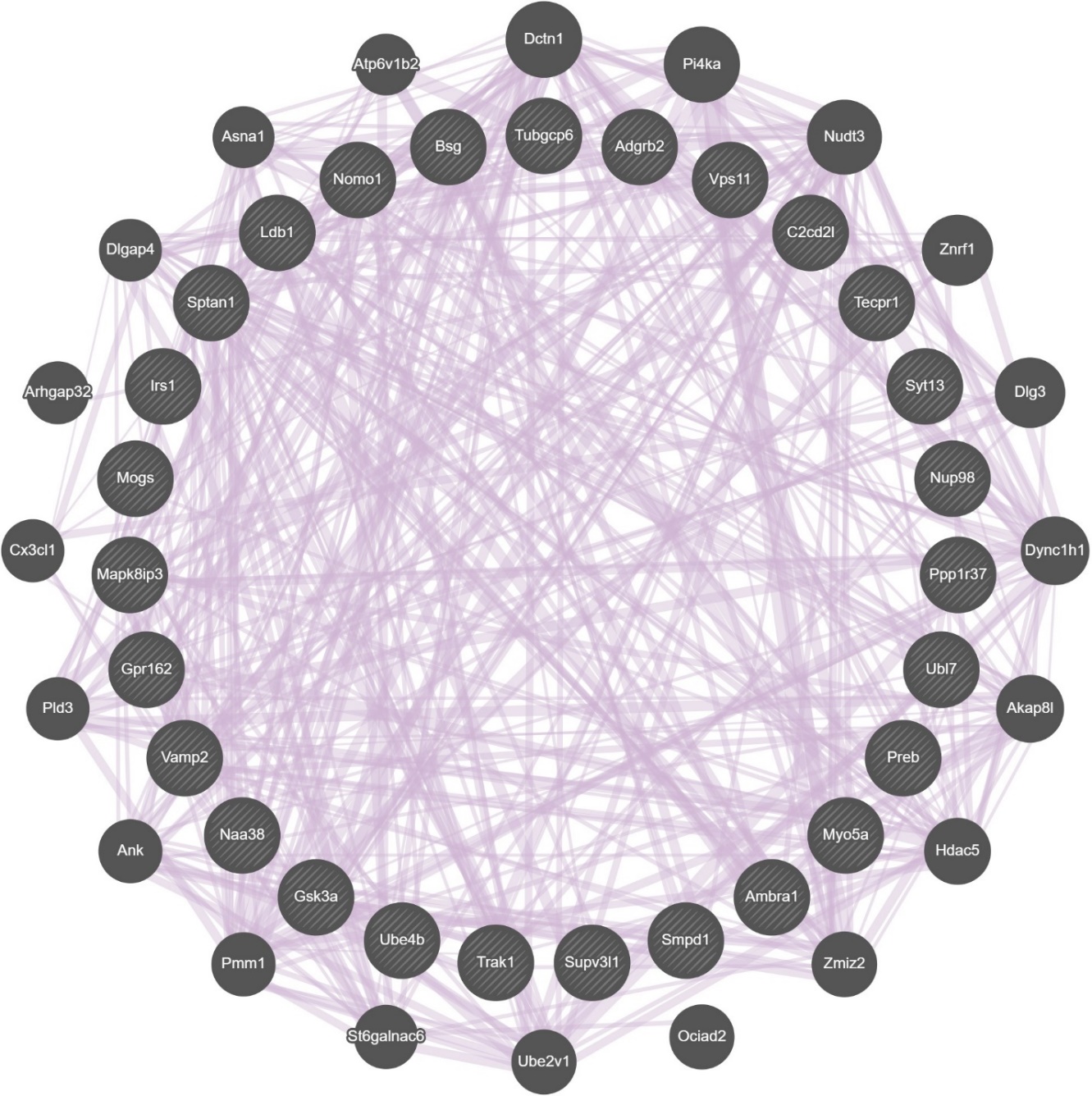


**Figure S1.** Gene–gene coexpression interaction network for differential splicing in the ventral midbrain (VMB), associated with relative genetic risk for MA intake. This network was derived from the 27 hub nodes (inner cycle; symbol size has no meaning) in the MALDR line that has an increase of ≥ 0.5 in intramodular connectivity. The genes in the outer circle were identified by GeneMANIA [32] as functional associated (symbol size determined by GeneMANIA, based on number of network connections). Only gene-gene coexpression interactions are noted in this figure (pink lines).


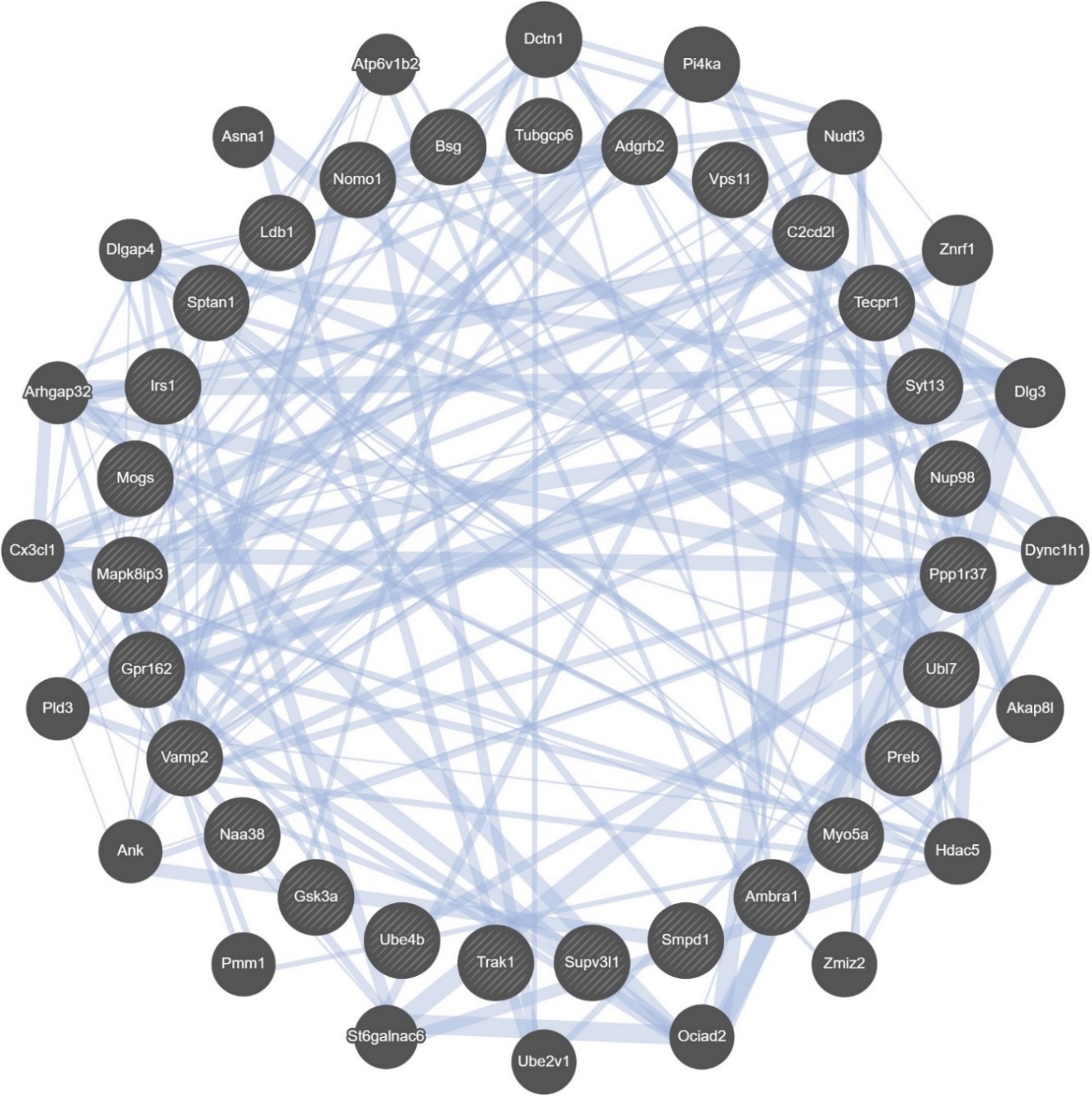


**Figure S2.** Gene–gene colocalization interaction network for differential splicing in the ventral midbrain (VMB), associated with relative genetic risk for MA intake. This network was derived from the 27 hub nodes (inner cycle; symbol size has no meaning) in the MALDR line that has an increase of ≥ 0.5 in intramodular connectivity. The genes in the outer circle were identified by GeneMANIA [32] as functional associated (symbol size determined by GeneMANIA, based on number of network connections). Only gene-gene colocalization interactions are noted in this figure (blue lines).


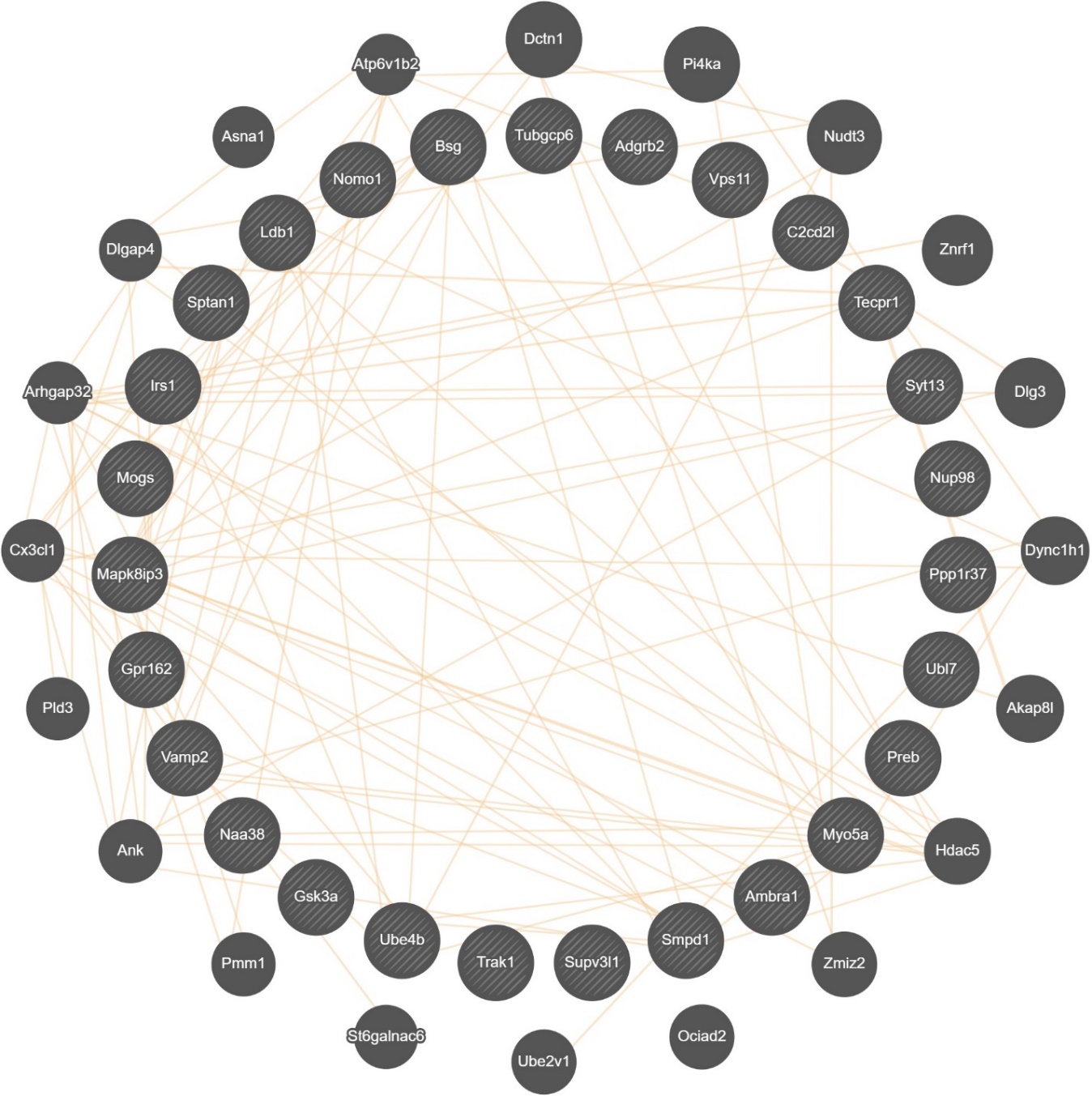


**Figure S3.** Gene–gene predicted interaction network for differential splicing in the ventral midbrain (VMB), associated with relative genetic risk for MA intake. This network was derived from the 27 hub nodes (inner cycle; symbol size has no meaning) in the MALDR line that has an increase of ≥ 0.5 in intramodular connectivity. The genes in the outer circle were identified by GeneMANIA [32] as functional associated (symbol size determined by GeneMANIA, based on number of network connections). Only predicted interactions are noted in this figure (tan lines).
